# EFFECTS OF ACTIVITIES ON THE PSYCHOLOGICAL WELL-BEING OF CAREGIVERS OF OLDER ADULTS

**DOI:** 10.1093/geroni/igad104.2523

**Published:** 2023-12-21

**Authors:** Sarah Won, Natalie Regier

**Affiliations:** Johns Hopkins University School of Nursing, Baltimore, Maryland, United States; Johns Hopkins University School of Nursing, Baltimore, Maryland, United States

## Abstract

It is well-known that the caregiving role is perceived as stressful and is often associated in the literature with decreased well-being. Given that approximately 41.8 million Americans provide unpaid care to an older adult, it is critical to identify strategies or interventions to maximize their well-being. Activity engagement is one such promising intervention. Consequently, the goal of this study was to investigate which activities of caregivers of older adults significantly improve their well-being. To that end, a three-stage literature review (title/abstract review, full-text review, reference list review) was conducted, resulting in a total of 2,959 articles identified in PsycINFO. Inclusion/exclusion criteria narrowed this down to 24 relevant full-text research articles. Results showed a total of eight cohesive categories of activities for caregivers: Physical (n=5), Social (n=5), Arts/entertainment in the home (n=2), Arts/entertainment outside of the home (n=2), Religious/Spiritual (n=4), Psychotherapy (n=5), Psychoeducation (n=3), and Multimodal (n=4). Indicators of caregiver well-being in the outcome variables of studies included depressive symptoms, overall mental health, stress-induced cellular aging, life satisfaction, distress, quality of life, caregiver burden, satisfaction with the caregiving role, salivary cortisol, caregiver mastery, and stress. Activity categories that had the greatest frequency of positively impacting caregiver well-being were physical activities, social activities, religious/spiritual activities, and psychoeducational activities. All other activity categories except for arts/entertainment activities outside of the home were effective in some studies in improving caregiver well-being. Findings suggest that caregivers of older adults should seek opportunities for engagement in meaningful activity, particularly physical, social, religious/spiritual, and psychoeducational activities.

